# Supplementary material for: TR-FRET between engineered nanobodies reveals the existence of endogenous CXCR4 oligomers
Source: Commun Biol. 2025 Dec 16;8:1777. doi: 10.1038/s42003-025-09166-6 (PMC12708788; doi:10.1038/s42003-025-09166-6)
Supplement: Supplementary file 1 — Supplementary information [file 42003_2025_9166_MOESM1_ESM.pdf]

Supplementary Information

TR-FRET between engineered nanobodies reveals the existence of endogenous CXCR4 oligomers

Joyce Heuninck[1] °, Vladimir Bobkov[2, 5] °, Claire M. Grison[1], Amos Fumagalli[1], Mathias Lescuyer[1], Omolade Otun[1], Cherine Bechara[1], Laurent Lamarque[3], Eric Trinquet[3], Françoise Bachelerie[4], Philippe Marin[1], Bernard Mouillac[1], Sebastien Granier[1], Bas van der Woning[2], Hans de Haard[2], Martine J. Smit[5], Raimond Heukers[5, 6] *, Thierry Durroux[1] *

° Both authors contributed equally to this work
* Corresponding authors

Affiliation:
[1] IGF, Université de Montpellier, CNRS, INSERM, Montpellier, France
[2] Argenx BVBA, Industriepark Zwijnaarde 7, 9052 Zwijnaarde, Belgium
[3] Revvity, Parc Marcel Boiteux – BP 84175, 30200 Codolet, France
[4] Inserm UMR996 - Inflammation, microbiome and immunosurveillance, , Université Paris-Saclay, Clamart, France
[5] Division of Medicinal Chemistry, Amsterdam Institute for Molecules, Medicines and Systems (AIMMS), VU University Amsterdam, De Boelelaan 1108, 1081 HZ, Amsterdam, The Netherlands
[6] QVQ Holding BV, Yalelaan 1, 3584 CL, Utrecht, The Netherlands

Supplementary figures

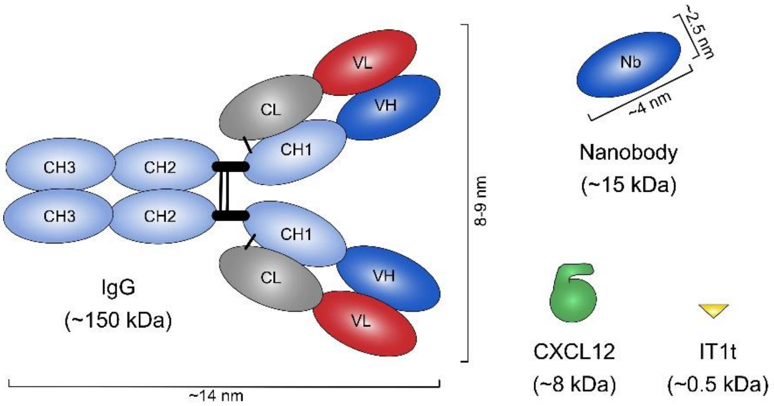

Figure S1. Schematic representation of molecules which are able to label CXCR4 and/or ACKR3 receptors: antibodies and nanobodies specific for either CXCR4 or ACKR3, CXCL12, the natural agonist for CXCR4 and ACKR3 and IT1t, a small antagonist specific for CXCR4. Although the representation does not consider the conformation of the molecules, it gives a relative proportion of each of them.

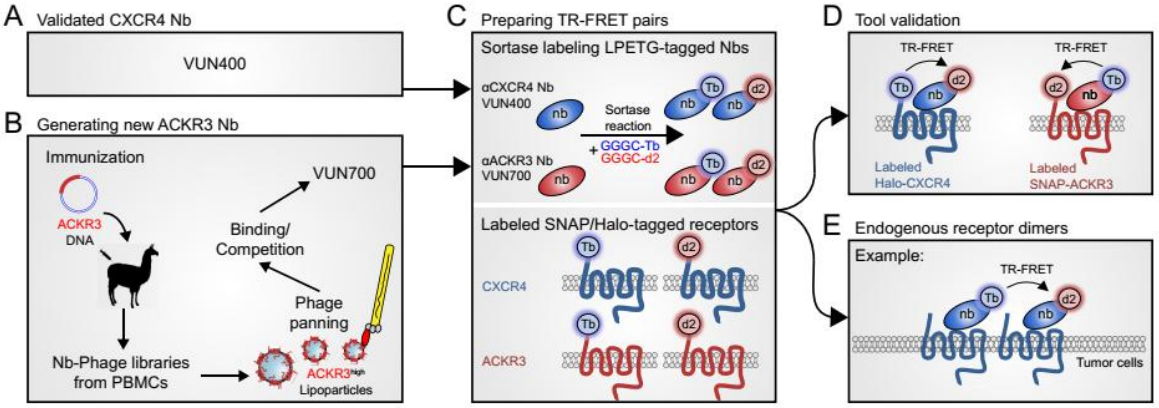

Figure S2. Schematic representation of the nanobody development, labeling and application workflow. A) CXCR4 nanobody VUN400 was isolated and characterized previously [1,2]. B) Llamas were immunized with ACKR3 cDNA followed by phage display selection on ACKR3 expressing lipoparticles and screening of individual clones for ACKR3 binding and CXCL12 displacement. C) VUN400 and VUN700 were modified to allow directional conjugation using the sortase method. Only the C-terminally labeled nanobodies are depicted. This involved the addition of an LPETG motif upstream a His-tag at the C-terminus of the nanobody, and subsequent labeling with either GGGC-lumi4-Tb or GGGC-d2 using the sortase transpeptidase method (top) [3]. In addition, CXCR4 and ACKR3 fused to a N-terminal SNAP- or Halo-tag were labeled with either Lumi4-Tb or d2 (bottom). D) TR-FRET between overexpressed labeled receptors in transfected cells and nanobodies labeled in both orientations was validated. E) Endogenous CXCR4 oligomers were detected in tumor cells using pairs of Lumi4-Tb- and d2-labeled nanobodies.

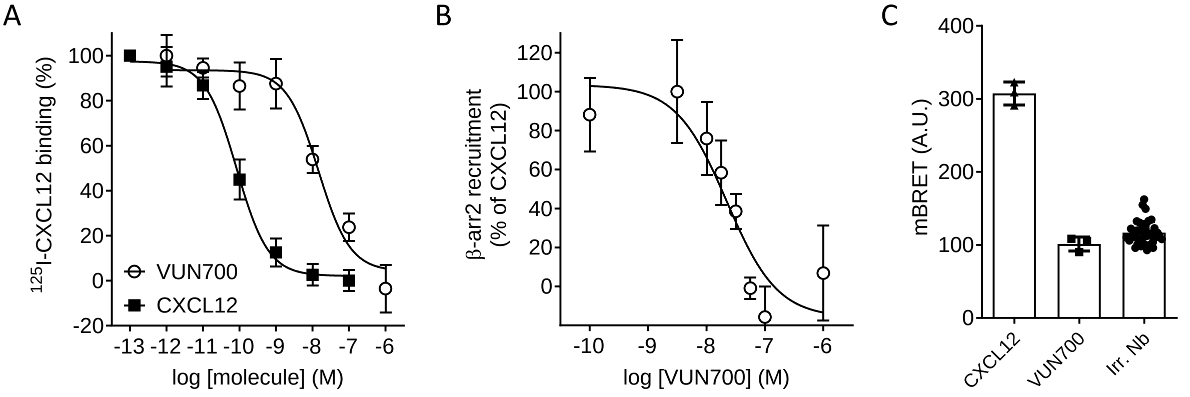

Figure S3. VUN700 displaces CXCL12 from ACKR3 and demonstrates antagonistic properties. A) Inhibition of ^{125}I-CXCL12 binding to ACKR3-expressing HEK293T membranes by increasing concentrations of VUN700 (open circles) or non-labeled CXCL12 (closed squares). Data were fitted using “Binding Competitive - one site- Fit LogIC50” subroutine in Prism. pK_i value is mean ± SD from n=3 (VUN700) or n=5 (CXCL12) independent experiments. B) Inhibition of CXCL12-induced β-arrestin 2 recruitment in HEK293T cells transfected with ACKR3-Rluc8 and β-arrestin2-mVenus plasmids and treated with increasing concentrations of VUN700. Values are expressed in percentage of maximal effect, where 100% and 0% correspond to the positive (no VUN700 treatment, only CXCL12) and negative (no Nb treatment, no CXCL12) controls, respectively. Means ± SD of values obtained in three

technical replicates are illustrated. pIC50 is mean ± SD from n=3 independent experiments. Data were fitted using one “Dose response stimulation – Log(agonist) vs. response (three parameters) subroutine in Prism. C) β-arrestin 2 recruitment in HEK293T cells transfected with ACKR3-Rluc8 and β-arrestin2 mVenus plasmids and treated with either 80 nM CXCL12, 1 µM VUN700 or an irrelevant nanobody (Irr. Nb) in mean ± SD with n=3.

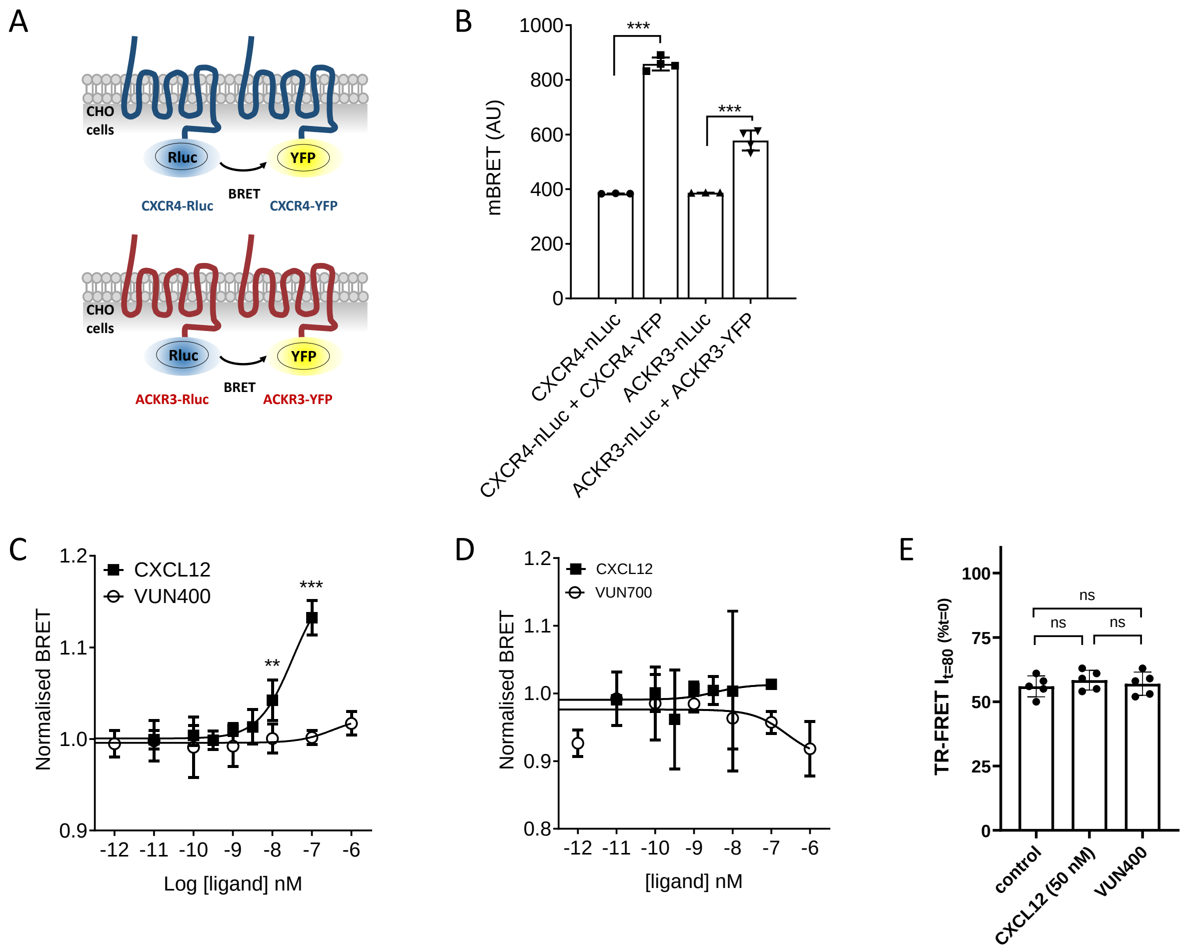

Figure S4. Ligand-induced change in BRET between CXCR4-nLuc and CXCR4-YFP or ACKR3-nLuc and ACKR3-YFP. A) Schematic representation of BRET between receptors. B) BRET between nLuc- and YFP-tagged CXCR4 or ACKR3. CHO-K1 cells were transfected with plasmids encoding either CXCR4-nLuc and CXCR4-YFP or ACKR3-nLuc and ACKR3-YFP or the nLuc-tagged receptors alone. Coelenterazine was used as a substrate for nLuc. The BRET signal was normalized to the nLuc signal. Mean ± SD, n=3. C) BRET signals between nLuc- and YFP-tagged CXCR4, 10 minutes after addition of increasing concentrations of either CXCL12 or VUN400. Mean ± SD, n=3. D) BRET signals between nLuc- and YFP-tagged ACKR3, 10 minutes after addition of increasing concentrations of CXCL12 or VUN700. Mean ± SD, n=2. Significance of change in BRET signal was determined by unpaired t-test (** $p < 0.01$ and *** $p < 0.001$). E) CHO-K1 cells expressing HALO-CXCR4 receptor were labeled with HALO-Lumi4-Tb (100 nM) and HALO-d2 (300 nM) substrates to get half of the receptors labeled with the fluorophore donor and half with the acceptor. After washing steps to remove the excess of unbound substrates (heterogenous conditions), cells were incubated with or without VUN400 (100 nM) or CXCL12 (50 nM). TR-FRET signal evolution was followed for 90 minutes. TR-FRET intensities measured at 80 minutes were expressed as % of TR-FRET

intensity at time 0. Mean ± SD n=5 independent experiments performed in hexaplicates or octaplicates.

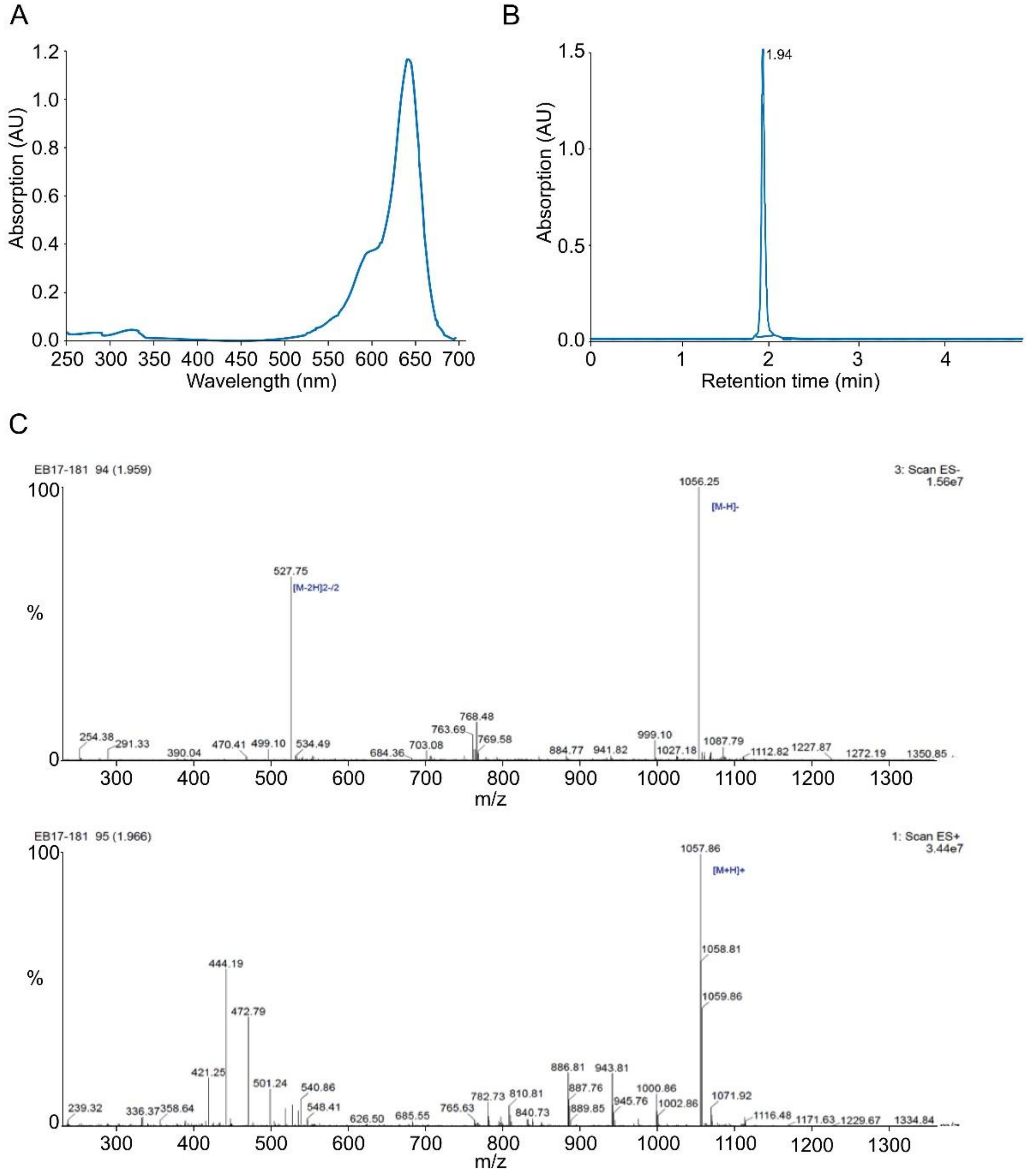

Figure S5. Spectra of GGGC-d2 peptide. A) UV-VIS spectrum, B) Chromatogram from high-performance liquid chromatography, C) nano-ESI mass spectrum.

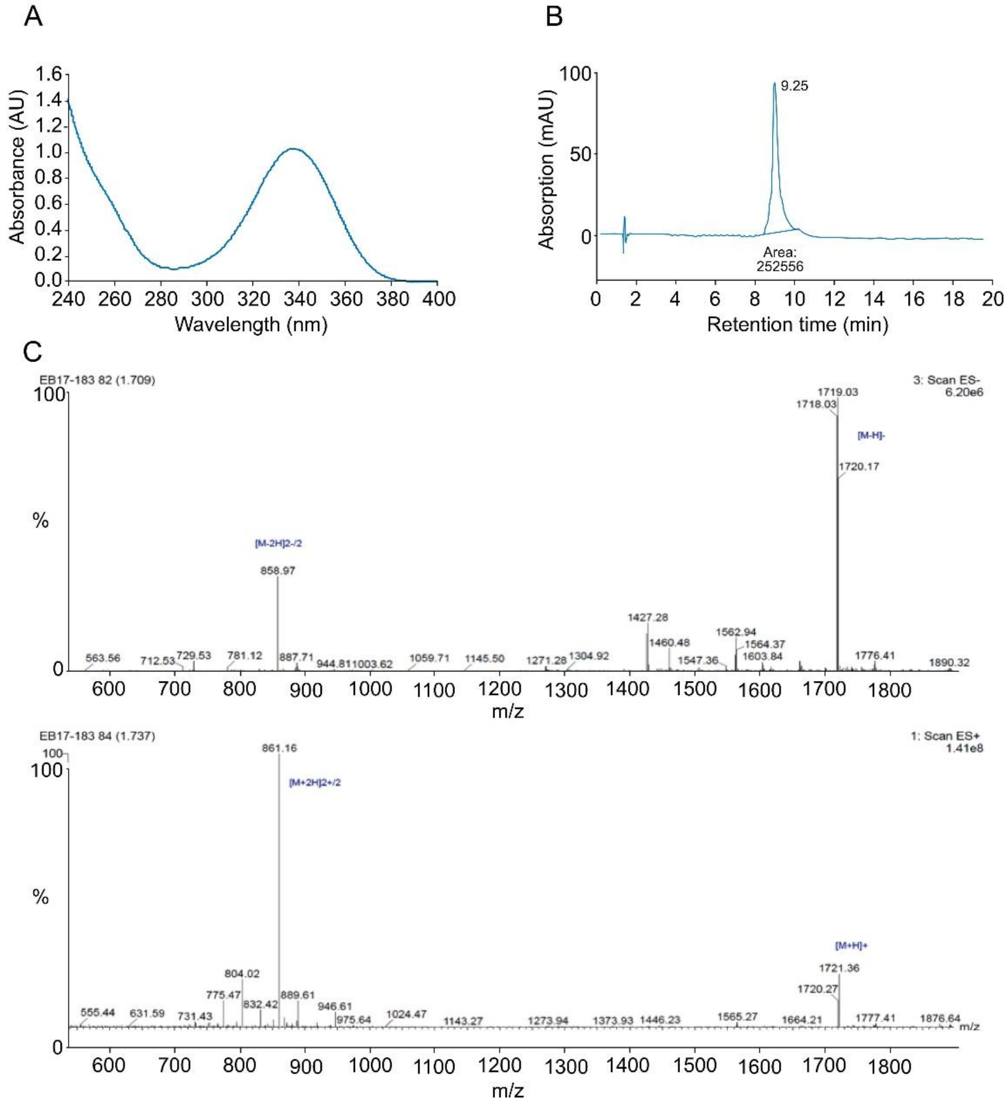

Figure S6. Spectra of GGGC-Tb peptide. A) UV-VIS spectrum, B) Chromatogram from high-performance liquid chromatography, C) nano-ESI mass spectrum.

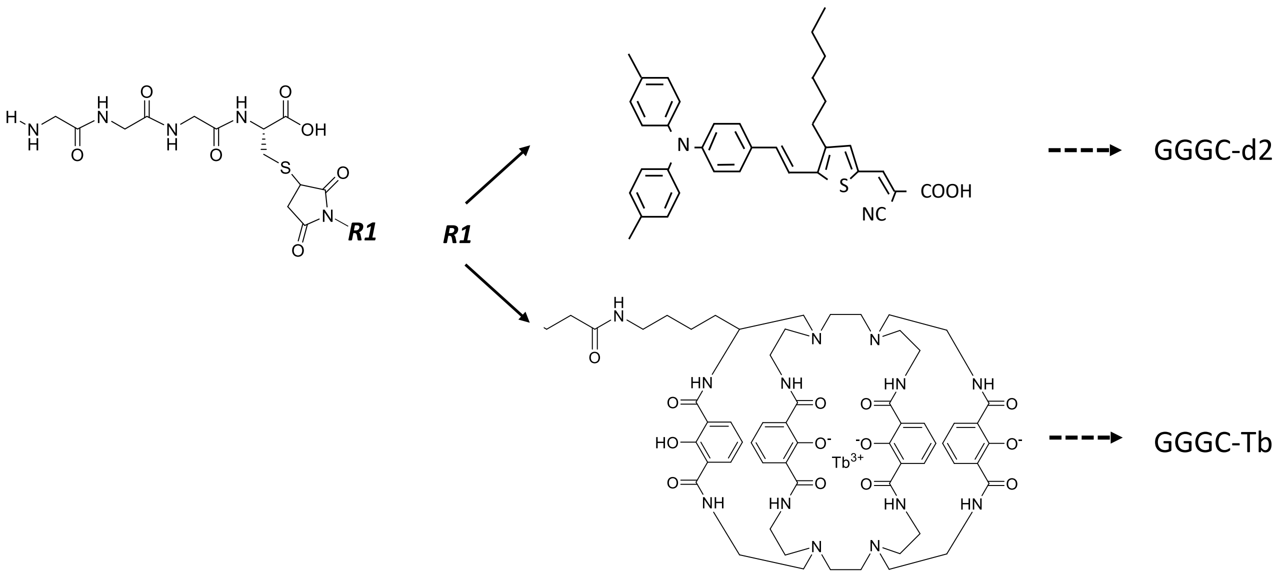

Figure S7. Structure of the peptides GGGC-d2 and GGGC-Tb.

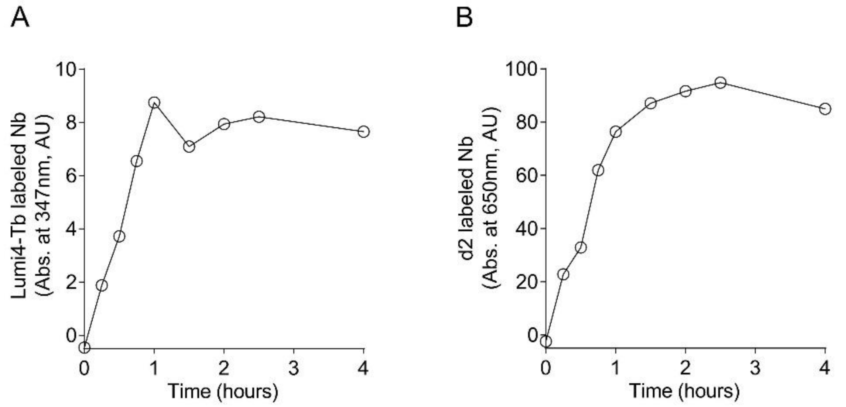

Figure S8. Kinetics of sortase mediated labeling. A) The amount of Lumi4-Tb-labeled VUN400 nanobody detected by absorbance at 347 nm in size-exclusion chromatography (SEC) was plotted over time. B) The amount of d2-labeled VUN400 nanobody detected by absorbance at 650 nm in SEC was plotted over time.

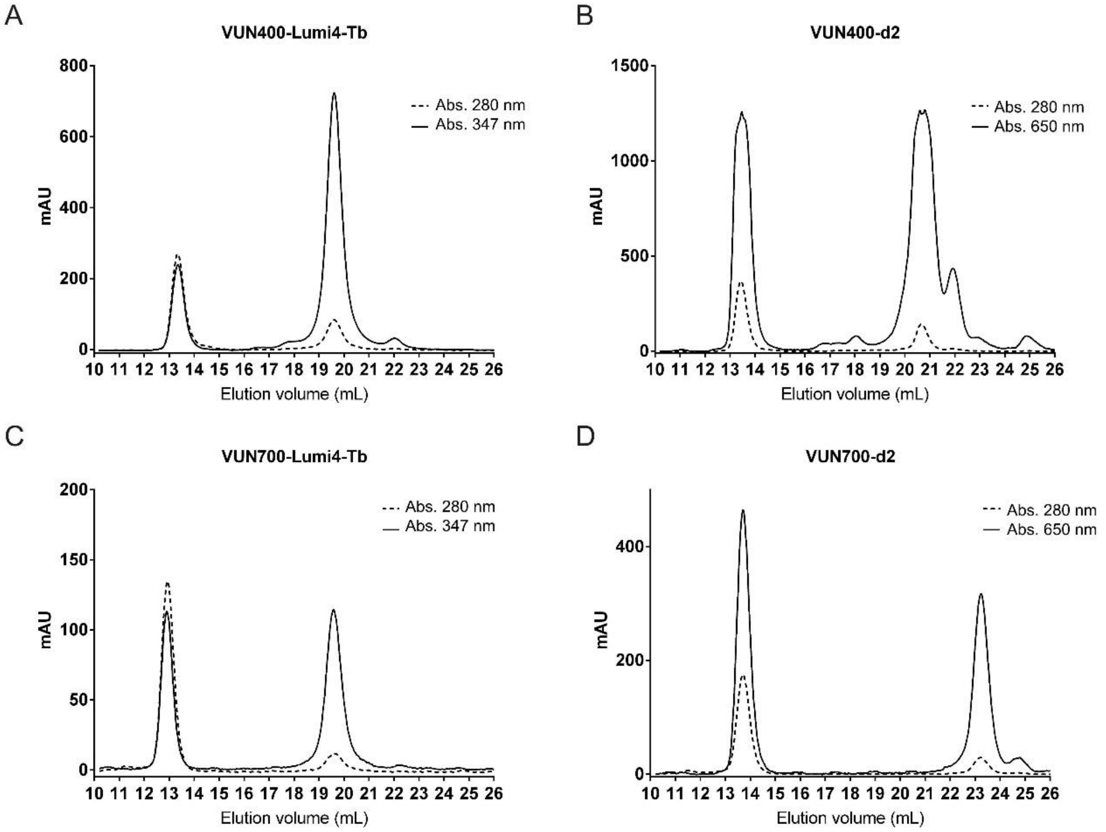

Figure S9. SEC chromatograms of labeled nanobodies. A) SEC chromatogram of VUN400 labeled with Lumi4-Tb. B) SEC chromatogram of VUN400 labeled with a d2 dye. C) SEC chromatogram of VUN700 labeled with Lumi4-Tb. D) SEC chromatogram of VUN700 labeled with d2. Two different wavelengths have been indicated: 280 nm corresponding to the absorbance of the protein, 347 nm to the absorbance of the donor molecule (Lumi4-Tb) and 650 nm to the absorbance of the acceptor (d2) molecule. The first peak corresponds to the labeled nanobody, while the second peak corresponds to the labeled peptide that did not react with the nanobody.

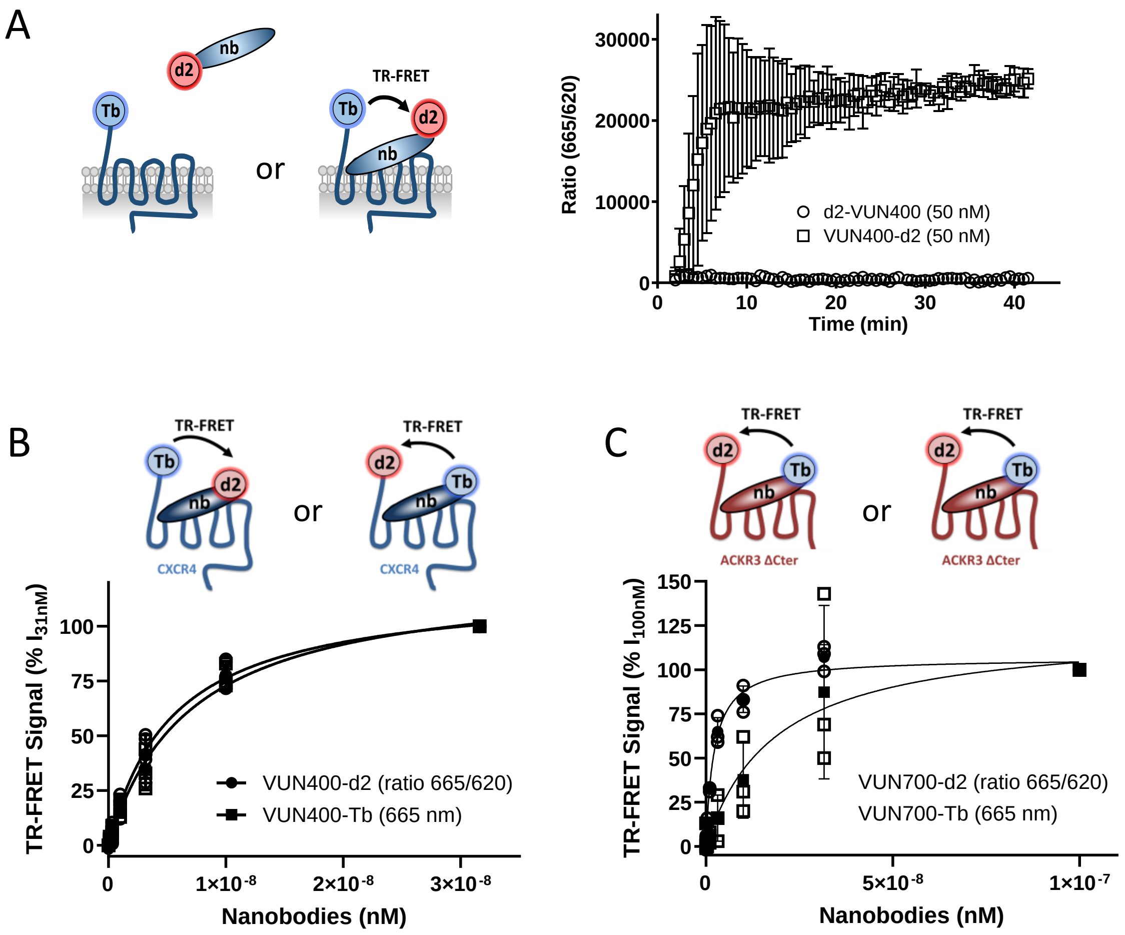

Figure S10. Detection of binding of labeled nanobodies to labeled receptors by TR-FRET. A) Nanobodies were either N- or C-terminally labeled with the d2 fluorophore using the sortase method, d2-VUN400 and VUN400-d2, respectively. CHO-K1 cells expressing Halo-CXCR4 were labeled with Halo-Lumi4-Tb. Binding of N-terminally labeled d2-VUN400 (circles) and C-terminally labeled VUN400-d2 (squares) to Lumi4-Tb-CXCR4 was followed over time by determining TR-FRET (ratio 665/620). Data presented as means ± SDs of values from one representative experiment performed in triplicate are shown. B) Binding (TR-FRET signal) of a concentration range of C-terminally Lumi4-Tb- or d2-labeled VUN400 to d2- (squares) or Lumi4-Tb- (circles) labeled Halo-CXCR4, respectively, in CHO-K1 cells. C) Binding experiments of a concentration range of C-terminally Lumi4-Tb- or d2-labeled VUN700 to d2- (squares) or Lumi4-Tb-SNAP-ACKR3-ΔCter (circles), respectively, in CHO-K1 cells. TR-FRET measurements have been performed after washing cell steps to remove the excess of the ligands. Lumi4-Tb was excited at 349 nm and the emission of the donor was measured at 620 nm. The TR-FRET signals were determined by measuring signal intensity at 665 nm (in case of Lumi4-Tb-labeled nanobodies) or by calculating the 665/620 nm intensity ratio (for d2-labeled nanobodies). The data represented are the means ± SDs of values obtained in three independent experiments.

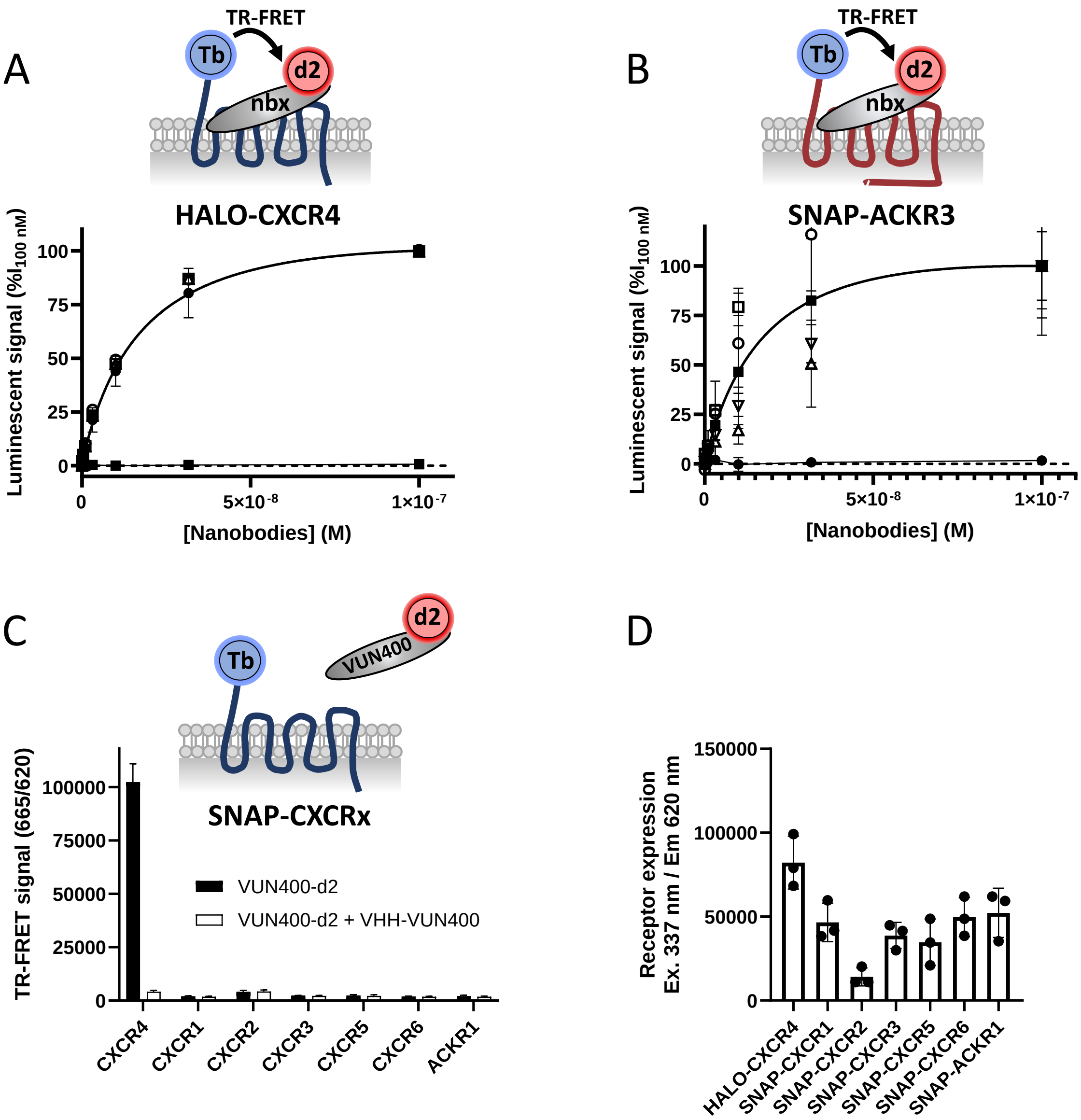

Figure S11: selectivity of VUN400 and VUN700. Because both CXCR4 and ACKR3 bind CXCL12 with a high affinity, the selectivity VUN400 and VUN700 for CXCR4 and ACKR3 was investigated. CHO-K1 cells were transfected to express HALO-CXCR4 or SNAP-ACKR3. Receptors have been labeled with Lumi4-Tb and saturation experiments were performed with VUN400-d2 and VUN700-d2 on HALO-CXCR4 (A) and SNAP-ACKR3 (B). Each open symbol correspond to one experiment performed in triplicate. Closed symbols correspond to the mean of 3 (A) or 4 (B) experiments. Data (% of the intensity at a concentration of 100 nm for the nanobodies) are expressed as means (open symbols) or means ± SDs (closed symbols). No specific binding could be observed with VUN 400-d2 and VUN700-d2 on SNAP-ACKR3 and HALO-CXCR4, respectively, proving the selectivity of VUN400 and VUN700 for their cognate receptors.

The selectivity of VUN400 was also investigated on chemokine receptors which can bind CXCLx chemokine (C). We expressed SNAP-CXCR1, SNAP-CXCR2, SNAP-CXCR3, SNAP-CXCR5, SNAP-CXCR6 or SNAP-ACKR1 receptors in HEK 293 (D). Receptors have been labelled with Lumi4-Tb and incubated in the presence of VUN400-d2 at a high concentration (100 nM). Non-specific binding of VUN400-d2 was determined in the presence of an excess of VHH-VUN400 (10 µM). HALO-CXCR4 was used as positive binding control.

No significant difference were observed for any receptor in the presence or the absence of VHH-VUN400. Means + SDs from three independent experiments, each performed in triplicate.

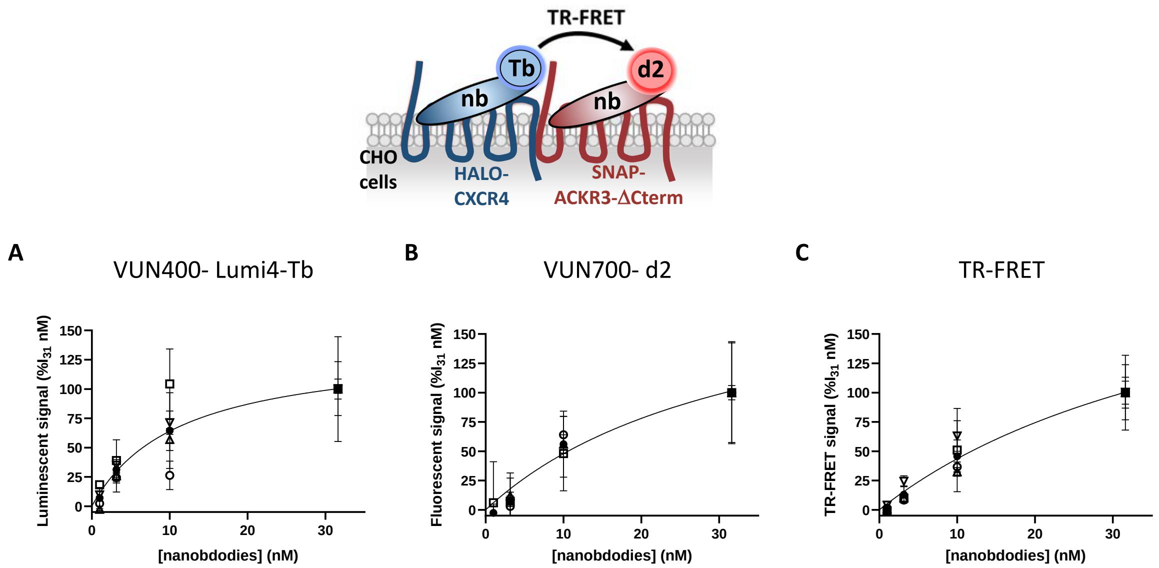

Figure S12. Detection of CXCR4 and ACKR3 hetero-oligomers in CHO transfected cells. Saturation and TR-FRET curves for VUN400-Lumi4-Tb (A) and VUN700-d2 (B) in CHO-K1 cells co-expressing Halo-CXCR4 and SNAP-ACKR3. For TR-FRET (C) both nanobodies were added to the cells at equal concentrations. Each open symbol corresponds to one experiment performed in triplicate. Closed circles correspond to the mean of the 3 different experiments. Data (% of the intensity at a concentration of the nanobodies of 31 nM) are expressed as means (open symbols) or means + SDs (closed circles). All values have been measured after performing washing steps to remove unbound nanobodies. The specific binding signal presented here was calculated by subtracting the non-specific binding signal, obtained in the presence of competitive molecules for CXCR4 (IT1t) and ACKR3 (unlabeled VUN700, CXCL12, and GD301), from the total binding signal. The emission bleed-through of Lumi4-Tb at the FRET wavelength has been subtracted. Means ± SDs of values obtained in three independent experiments are represented.

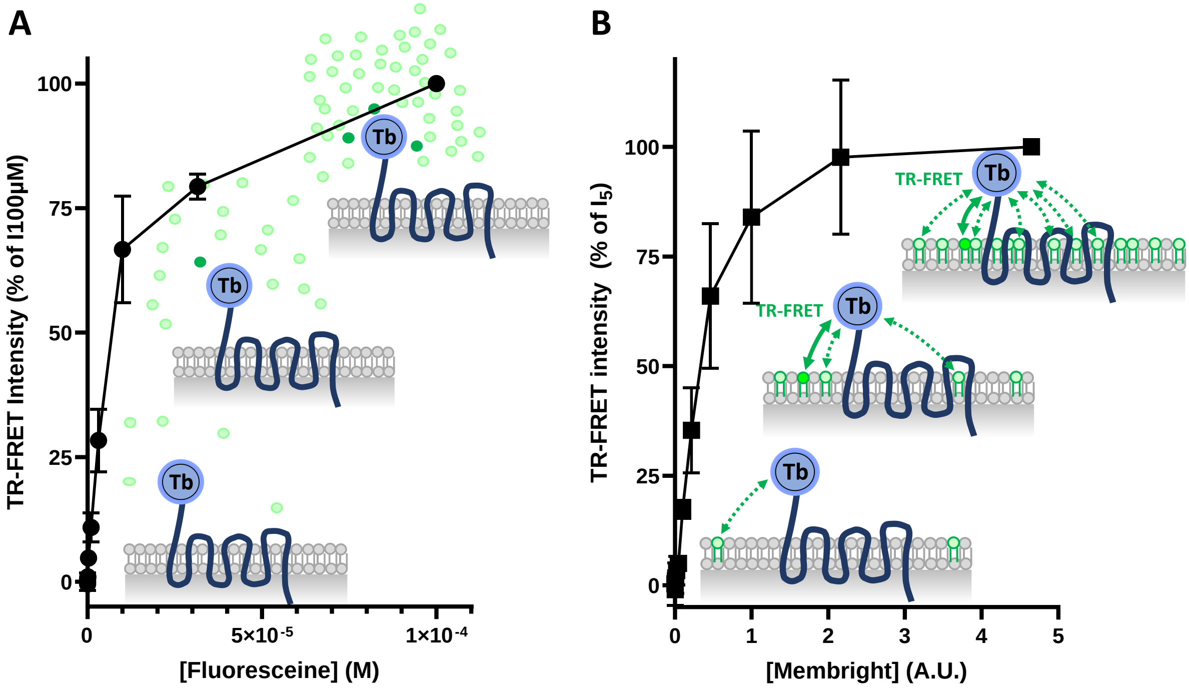

Figure S13: Variation of TR-FRET signal resulting from random collisions between labelled partner follows a parabolic curve. Two sets of experiments were performed to study the variation of TR-FRET signal resulting from a model of pure random collisions between two partners. In a first set of experiments, transfected CHO-K1 cells expressing HALO-CXCR4 were labelled with Lumi4-Tb and incubated in the presence of increasing concentration of fluorescein (green circle) in the extracellular medium (A). In a second set of experiments, transfected CHO-K1 cells expressing HALO-CXCR4 were labelled with Lumi4-Tb and incubated in the presence of increasing concentration of MemBright 488 (green circle), a fluorescent dye which is incorporated into the membrane (B). (concentration of 1 corresponds to the recommended concentration by supplier) TR-FRET signal (520 nm) was plotted as a function of dye concentration. Data were expressed as mean + SD were obtained from three independent experiments performed in quadruplicate.

Both plots illustrated that TR-FRET signal does not vary linearly in function of the concentration of the partners and the TR-FRET efficiency is dependent on the concentrations of the partner.

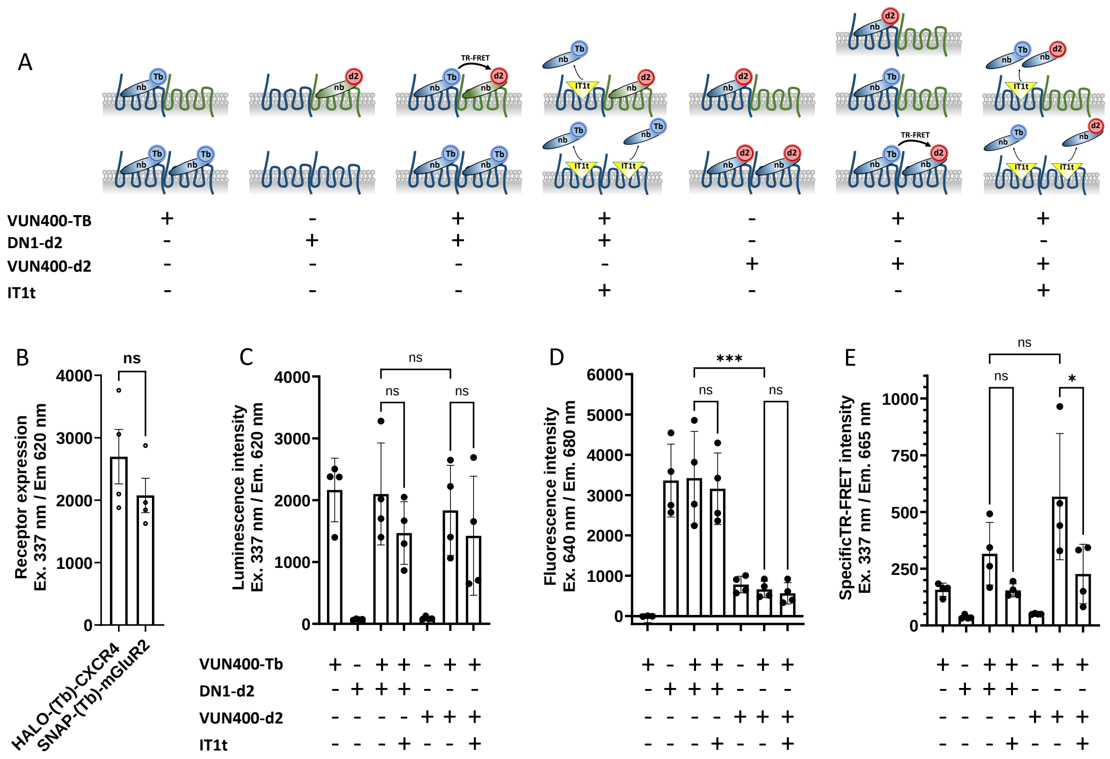

Figure S14: Transfected CHO cells similarly expressing HALO-CXCR4 and SNAP-mGLUR2 receptors are incubated in the presence of a mix of CXCR4-binding nanbodies VUN400-Tb, VUN400-d2 and mGluR2-binding nanobody DN1-d2[4]. Donor (C), acceptor (D) and TR-FRET (E) luminescence signals are shown. Despite binding of VUN400-Tb (C) and DN1-d2 (D), no significant specific TR-FRET signal was observed for this condition (E). By contrast, even at much less intense labeling with VUN400-d2 than with DN1-d2 (D), specific TR-FRET was observed between VUN400-Tb and VUN400-d2 (E). This suggests that the observed TR-FRET signals are due to interactions between CXCR4 receptors and not to random collisions between receptors. Furthermore, IT1t reduced the TR-FRET signals between labeled CXCR4, but not between CXCR4 and mGluR2, suggesting specificity of the detected CXCR4 interactions. A) Depiction of the different complexes of labelled CXCR4 (blue receptor) and mGlurR2 (green receptor) potentially observable upon binding of the different nanobodies (VUN400-Tb, VUN400-d2 and DN1-d2) and/or IT1t. B) Determination of the relative HALO-CXCR4 and SNAP-mGluR2 expression after labelling of HALO-CXCR4 or SNAP-mGluR2 receptors with HALO-Lumi4-Tb or SNAP-Lumi4-Tb substrates, respectively. Luminescence signals acquired at different wavelengths: donor luminescence: (ex: 337 nm; em: 620 nm) (C); acceptor fluorescence: ex: 645 nm; em: 680 nm (D); TR-FRET signal: ex: 337 nm; em: 665 nm (E). Nanobodies were used at near-saturating concentrations, corresponding to about 5xKd: VUN400-Tb: 125 nM, VUN400-d2: 100 nM; DN1-d2: 100 nM. IT1t was used at 5 µM. Four independent experiments were performed in triplicate. Bars correspond to means ± SDs. Statistics were performed using a one-way ANOVA analysis. *: $p < 0.1$; **: $p < 0.01$; ***: $p < 0.001$; ****: $p < 0.0001$.

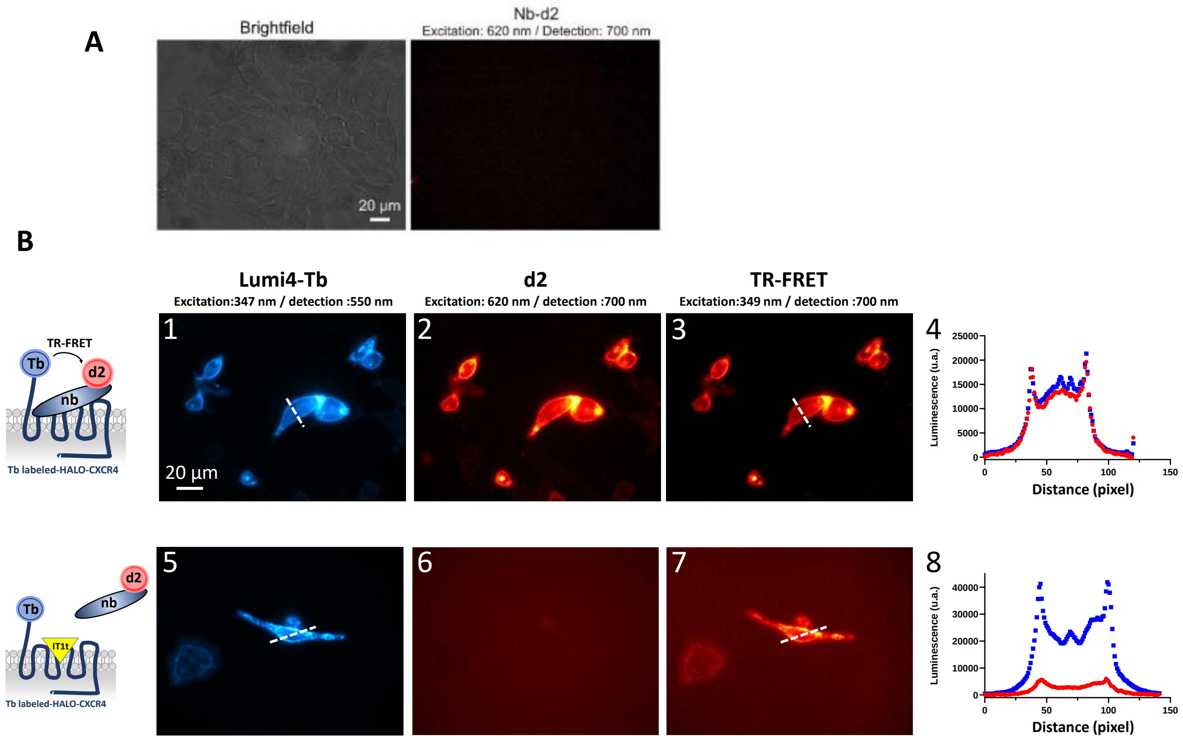

Figure S15. A significant TR-FRET signal depends on the binding of nanobodies to receptors. A) Negative controls for nanobody-receptor TR-FRET microscopy experiments. Mock HEK293T cells were incubated with VUN400-d2 for 2 h at 37°C. A brightfield image and a d2 signal image were taken using the TR-FRET microscope. B) HEK293T cells transfected with the Halo-CXCR4 construct were incubated with the Halo-Lumi4-Tb substrate. Labelled cells were then incubated with VUN400-d2 in the absence (1, 2, 3) or presence (5, 6, 7) of excess IT1t (2µM). Histograms (4, 8) correspond to the amplitude of donor and TR-FRET luminescence along the dotted white lines in the absence or presence of excess IT1t. The TR-FRET signal in the presence of IT1t is about 13% of the donor signal at 620 nm and corresponds mainly to terbium bleed through in the acceptor channel and probably also to random collisions of d2-labelled nanobodies of the medium with lumi4-Tb-labelled receptors.

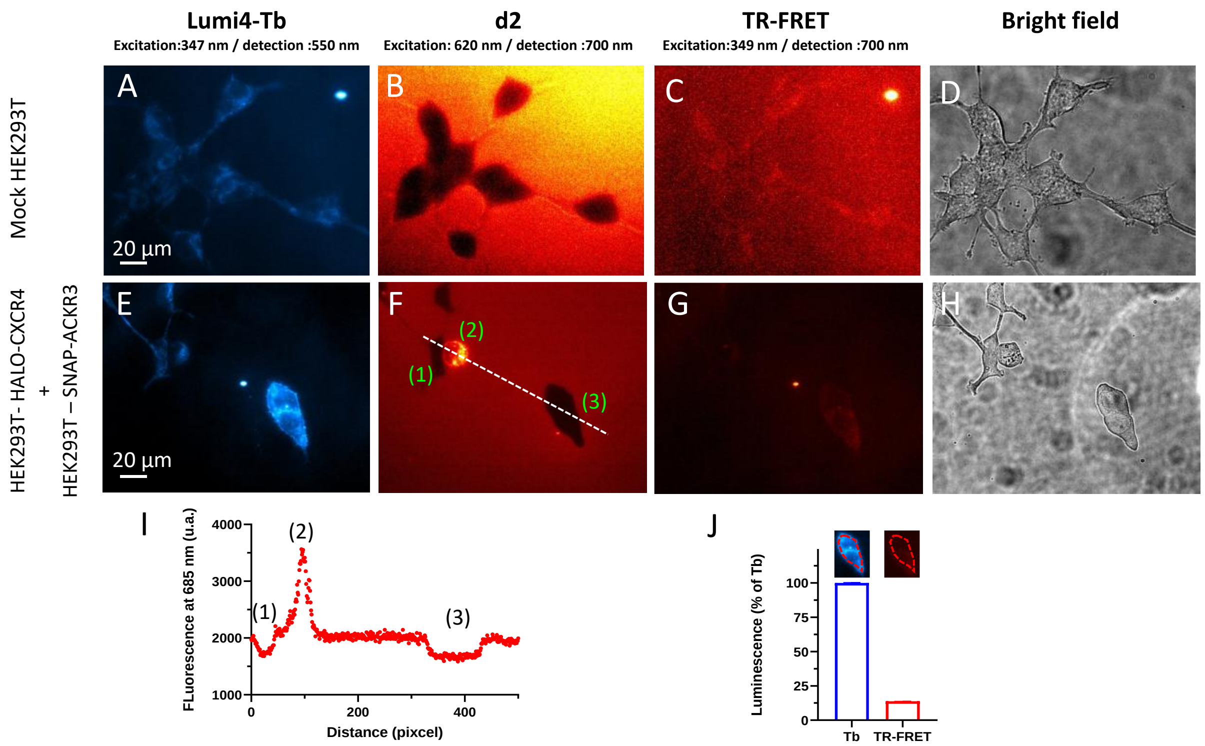

Figure S16: Nanobodies do not diffuse or weakly into the cells. HEK293T mock cells were incubated in the presence of VUN400-d2 (100 nM) and VUN700-Lumi4-Tb (100 nM). Donor (A), acceptor (B) and TR-FRET (C) luminescence signals were imaged. Weak non-specific luminescence labelling was observed in the donor and TR-FRET images (A and C). In contrast, the fluorescence image of the acceptor (B) showed a dark area corresponding to the cell silhouettes (D), indicating a weaker fluorescence signal in the cells than in the incubation medium. This is a strong confirmation that the nanobodies are not able to diffuse into the cells or that their penetration is weak. We performed similar experiments on HEK293T cells transfected with plasmids encoding SNAP-ACKR3 or HALO-CXCR4. Cells were plated in the same wells and incubated in the presence of VUN700-Lumi4-Tb (100nM) and VUN400-d2 (100nM). Donor luminescence (E), acceptor luminescence (F) and TR-FRET signal (G) were measured. Panel H corresponds to the brightfield image. The image corresponding to the acceptor luminescence (B) shows a cell positively labelled by VUN400-d2. In contrast, the cell positively labelled with VUN700-Lumi4-Tb (E) appears as a black area (F), again showing much weaker fluorescence than the medium containing VUN-400-d2. This is confirmed by the histogram (I), which corresponds to the plot of fluorescence intensity along the dashed white line. We measured donor and TR-FRET luminescence on a region of interest corresponding to the silhouette of the cell expressing ACKR3 (J). The intensity of the FRET signal corresponds to about 13% of the donor intensity at 620 nm. This is mainly due to terbium bleed-through in the acceptor channel and probably also to random collisions of d2-labelled nanobodies of the medium with lumi4-Tb-labelled receptors.

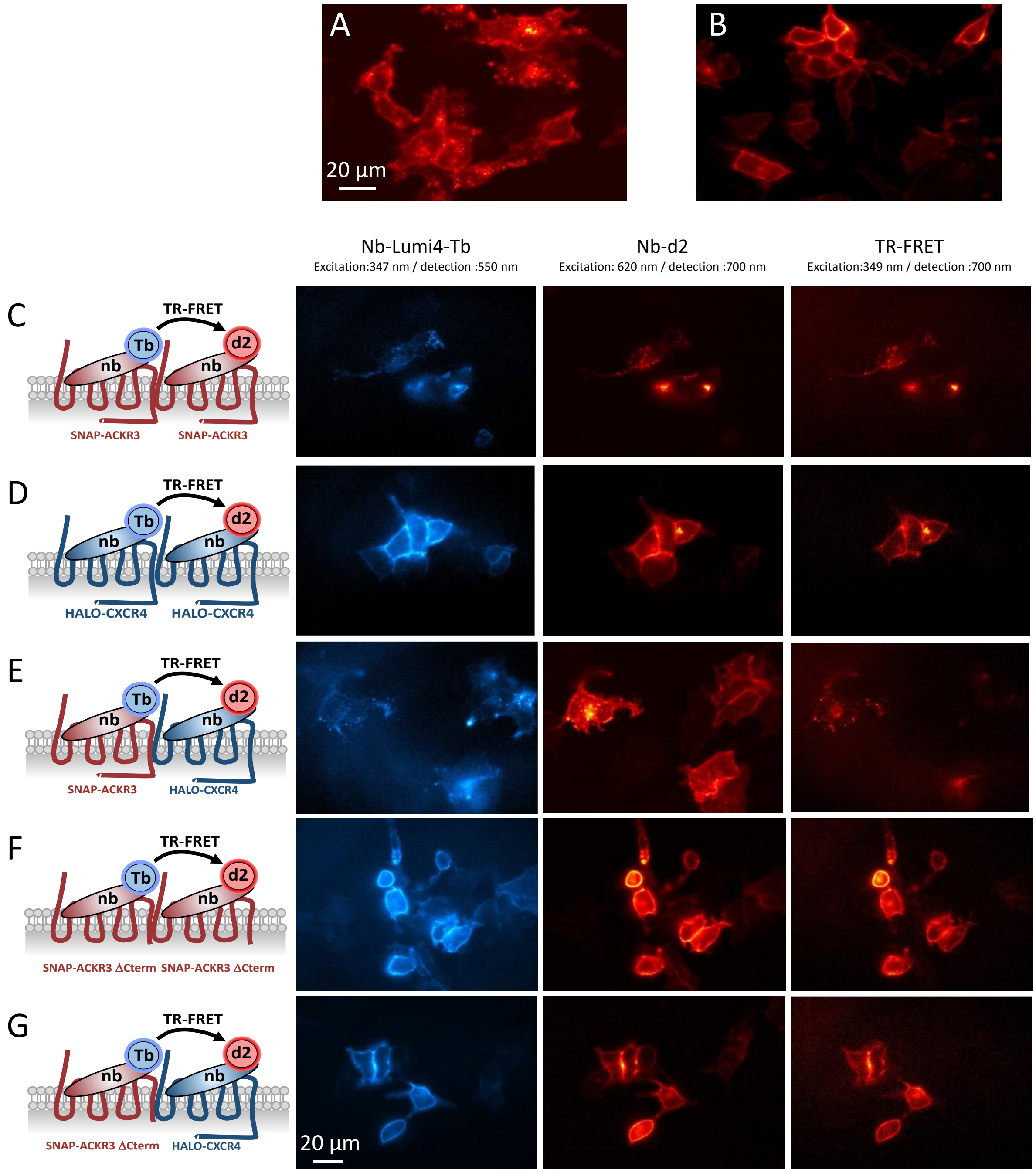

Figure S17: TR-FRET analysis of CXCR4 and wild type and C-terminally truncated ACKR3. We compared the labeling of SNAP-ACKR3 and SNAP-ACKR3-ΔCterm which has been reported to impaired constitutive internalization of ACKR3 [5], with VUN700-d2 (A and B). SNAP-ACKR3 labeling is heterogenous: it exhibits a labeling at the cell surface and a punctuated one in accordance with its constitutive internalization. By contrast, ACKR3-ΔCterm labeling is mainly located at the cell surface, as expected. Each of these receptors were then co-expressed with HALO-CXCR4. Luminescent signals of donor, acceptor and TR-FRET were imaged for all the conditions tested. As expected, donor, acceptor and TR-FRET luminescence of SNAP-ACKR3 showed a punctuated labeling corresponding to the constitutive internalization of the receptor (C). It can be associated to a labeling located at the cell surface on some cells. In contrast, HALO-CXCR4 and ACKR3-ΔCterm receptors labeled with donor and acceptor were mainly located at the cell surface (D, E, F and G). The TR-FRET

signal was therefore observed at the cell surface (C, F and G) except for HALO-CXCR4/SNAP-ACKR3 hetero-oligomers (E). For this latter case, labeling can be more heterogenous from cell to cell depending on the density of each receptor which affects their capacity to form either hetero or homo-oligomers. Nevertheless, the presence of punctuate labeling was expected, given the constitutive internalization of ACKR3.

Table S1. Wavelengths for fluorescent signal acquisition

Device	Wavelength (excitation/emission - nm)		
	Donor (Lumi4-Tb)	Acceptor (d2)	FRET
Plate-reader	337/620	645/680	337/665
TR-FRET microscope	349/550	620/700	349/700

Table S2. Affinity of fluorescently labeled VUN400 and VUN700 for labeled CXCR4 or ACKR3 in transfected CHO-K1 cells assessed by a saturation TR-FRET binding assay. The TR-FRET assay was performed with labeled Halo-CXCR4 or SNAP-ACKR3-ΔCterm and increasing concentrations of their respective nanobody in a heterogenous manner. TR-FRET signal between a receptor and nanobody was measured. pKd values represented in the table are the means ± SD of values obtained in three independent experiments. Nd : not determinable

	pKd Halo-CXCR4	pKd SNAP-ACKR3-ΔCterm
**VUN400-Lumi4-Tb**	8.5 ± 0.36	-
**VUN400-d2**	7.5 ± 0.26	nd
**VUN700-Lumi4-Tb**	-	7.8 ± 0.27 nM
**VUN700-d2**	nd	7.75.2 ± 0.15 nM

Binding curve modeling

We used a simplified theoretical model derived from the one we previously established to model binding curve when considering a monomer/dimer equilibrium (Durroux et al, TiPS 2005). To simplify the model, we made three hypotheses: i) nanobody labelling does not impact nanobody affinities for the receptor. Therefore, the nanobodies labeled either with Lumi4-Tb or d2 have the same affinity; ii) we considered that the affinity of a nanobody on a monomeric receptor is equal to the one of the first nanobody on a dimeric form (α=1 in the

table below); iii) we considered that both labelled nanobodies were added at the same concentration, as we did in the experimental conditions corresponding to Figure 1.

The results presented below correspond to four different cases:

- **A**: we postulated that two nanobodies can bind to a dimer with the same affinity (no positive or negative cooperative binding) (see table A below: α=β=γ=1). We fixed the affinity to 5 nM (approximatively the affinity we determined for the nanobodies with the Tag lite assay). Moreover, the constant of equilibrium between monomer and dimer (Kdim) was set to 0.1 nM. At this value, the proportion of monomer in the absence of nanobodies is about 98% of the total receptor.
- **B**: we postulated that there is a positive cooperative binding of nanobodies on a dimer. We considered a cooperative factor of 0.1. It means that the affinity of a second nanobody is 10 times better when a first nanobody is already bound to the receptor (see Table A: α=1; β=γ=0.1). As in condition A, Kdim was set at 0.1 nM. Of note, positive cooperativity in the binding has been reported for antagonists on different receptors (for example see [6]).
- **C**: we kept a positive cooperativity for the binding of a second nanobody when a first nanobody is already bound and we set Kdim at 0.01 nM, meaning that in the absence of nanobodies, the proportion of monomer is about 85% of the total receptor.
- **D**: we kept a positive cooperativity for the binding of a second nanobody when a first nanobody is already bound and we set Kdim at 0.001 nM, meaning that in the absence of nanobodies, the proportion of monomer is about 50% of the total receptor.

All parameter values used for the models are indicated in the table below.

	**A**	**B**	**C**	**D**
Rt (M)	1 E-12	1 E-12	1 E-12	1 E-12
Kdim (M)	1E-10	1E-10	**1E-11**	**1 E-12**
Kd (M)	5 E-9	5 E-9	5 E-9	5 E-9
α	1	1	1	1
β	1	**0.1**	**0.1**	**0.1**
γ	1	**0.1**	**0.1**	**0.1**

With Kdim = $[R]^2/[RR]$

Kd = [N1][R]/[RR] = [N2][R]/[RR]

αKd = [N1][RR]/ [N1RR] = [N2][RR]/ [N2RR]

βKd = [N1][N1RR]/ [N1RRN1] = [N1][RRN1]/ [N1RRN1]

= [N2][N2RR]/ [N2RRN2] = [N2][RRN2]/ [N2RRN2]

γKd = [N2][N1RR]/ [N1RRN2] = [N2][RRN1]/ [N2RRN1]

Rt : total receptor (1e-12 M); N1: Nanobody 1; N2: Nanobody 2; Kdim : dissociation constant of dimer, α, β and γ correspond to the cooperative coefficients for the binding of nanobodies on the different dimer/nanobody complexes.

The different binding curves are illustrated in the figure below. Insets correspond to a zoom on the feet of the curves.

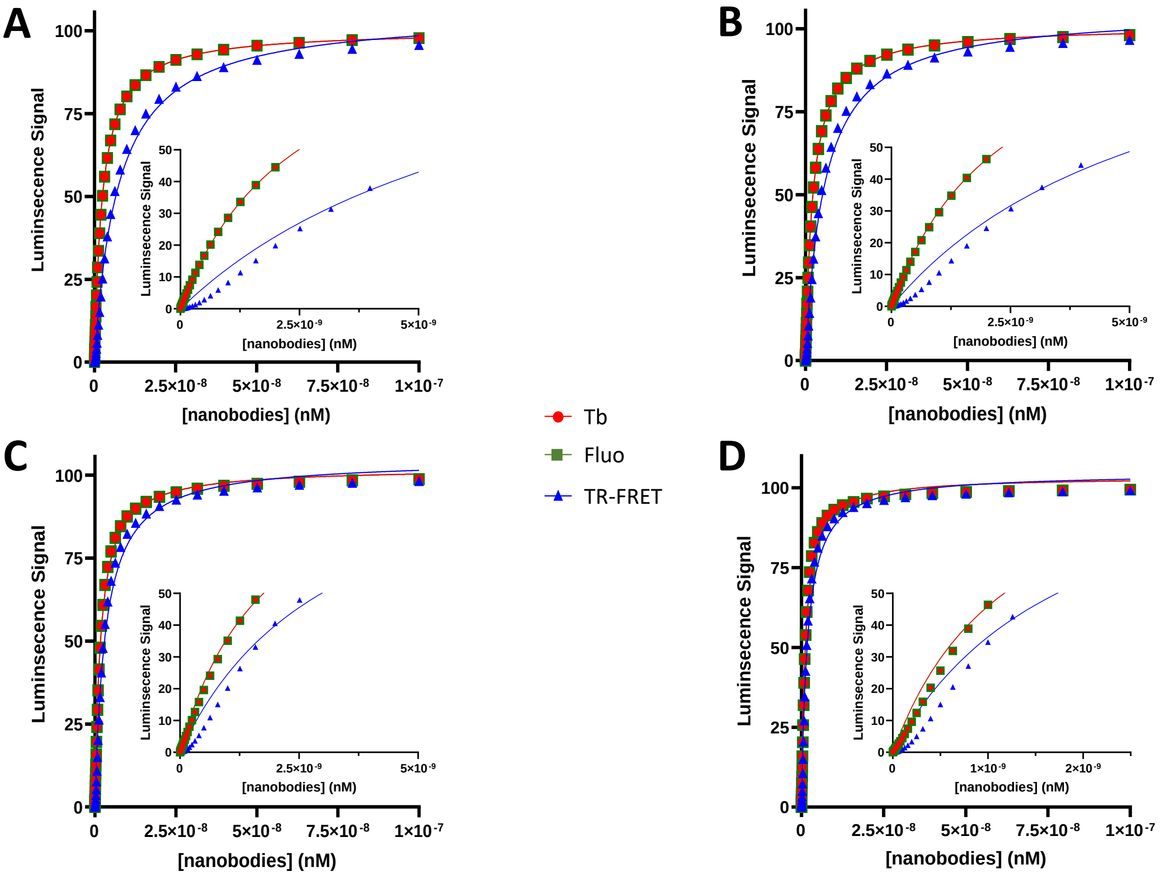

Figure S18: curves were fitted when considering one-site saturation equation in Prism although we are aware that it does not exactly correspond to the experimental conditions used for Figure 1 of the manuscript. EC50 values were reported in the table below.

	EC50		
	**Tb**	**D2 fluorescence**	**TR-FRET**
**A**	2.50	2.50	7.30
**B**	2.36	2.36	5.84
**C**	1.83	1.83	3.26
**D**	1.25	1.25	1.88

In condition A, we observed a right shift of the TR-FRET curve; the EC50 for TR-FRET signal is about 3 times higher than for those of Tb and d2 fluorescence signals. The differences between the EC50s are decreased when considering a positive cooperative binding on dimeric receptor (condition B) et even more when considering the presence of a significant percentage of dimer in the absence of nanobodies (Conditions C and D).
Moreover, all the insets clearly show sigmoid curves but sigmoidity of the curves can be observed only on a narrow range of nanobody concentration.

In the present study , we reported that for ACKR3 the EC50 for TR-FRET signal is about twice of the EC50s for Tb and d2 fluorescence. This is close to what we observed in condition B and C. Moreover, for CXCR4, we observed superimposed curves. This could suggest the presence

of a substantial % of dimeric receptors in the experimental conditions used to express CXCR4 receptors (high expression of HALO-CXCR4 in CHO or HEK 293 cells). This would be in accordance with the results reported by M. Lohse research group which has reported that when CXCR4 were expressed at high concentration, CXCR4 homodimers became prevalent (Isbilir et al, PNAS 2023).

Regarding the sigmoidity of the curves, because it can only be observed on a narrow range of nanobody concentrations, it is almost impossible to observe it with the Tag lite binding assay.

References

1. Van Hout, A. *et al.* CXCR4-targeting nanobodies differentially inhibit CXCR4 function and HIV entry. *Biochem. Pharmacol.* **158**, 402–412 (2018).
2. Bobkov, V. *et al.* Nanobody-Fc constructs targeting chemokine receptor CXCR4 potently inhibit signaling and CXCR4-mediated HIV-entry and induce antibody effector functions. *Biochem. Pharmacol.* **158**, 413–424 (2018).
3. Popp, M. W.-L., Antos, J. M. & Ploegh, H. L. Site-specific protein labeling via sortase-mediated transpeptidation. *Curr Protoc Protein Sci* **Chapter 15**, Unit 15.3 (2009).
4. Scholler, P. *et al.* Allosteric nanobodies uncover a role of hippocampal mGlu2 receptor homodimers in contextual fear consolidation. *Nat Commun* **8**, 1967 (2017).
5. Ray, P. *et al.* Carboxy-terminus of CXCR7 regulates receptor localization and function. *Int J Biochem Cell Biol* **44**, 669–678 (2012).
6. Mattera, R., Pitts, B. J., Entman, M. L. & Birnbaumer, L. Guanine nucleotide regulation of a mammalian myocardial muscarinic receptor system. Evidence for homo- and heterotropic cooperativity in ligand binding analyzed by computer-assisted curve fitting. *J. Biol. Chem.* **260**, 7410–7421 (1985).
7. van der Woning, B. *et al.* DNA immunization combined with scFv phage display identifies antagonistic GCGR specific antibodies and reveals new epitopes on the small extracellular loops. *MAbs* **8**, 1126–1135 (2016).
8. Bobkov, V., van der Woning, B. & de Haard, H. Display Technologies for Generation of Ig Single Variable Domains. *Methods Mol. Biol.* **1827**, 129–144 (2018).

9. Proft, T. Sortase-mediated protein ligation: an emerging biotechnology tool for protein modification and immobilisation. *Biotechnol. Lett.* **32**, 1–10 (2010).

10. Chen, I., Dorr, B. M. & Liu, D. R. A general strategy for the evolution of bond-forming enzymes using yeast display. *Proc. Natl. Acad. Sci. U.S.A.* **108**, 11399–11404 (2011).
