# Supplementary material for: TR-FRET between engineered nanobodies reveals the existence of endogenous CXCR4 oligomers
Source: Commun Biol. 2025 Dec 16;8:1777. doi: 10.1038/s42003-025-09166-6 (PMC12708788; doi:10.1038/s42003-025-09166-6)
Supplement: Supplementary file 2 — Description of Additional Supplementary Files [file 42003_2025_9166_MOESM2_ESM.pdf]

# Description of Additional Supplementary Files

**File name:** Supplementary Data

**Description:** the data supporting the findings of this study
